# A New Breviane Spiroditerpenoid from the Marine-Derived Fungus *Penicillium* sp. TJ403-1

**DOI:** 10.3390/md16040110

**Published:** 2018-03-29

**Authors:** Beiye Yang, Weiguang Sun, Jianping Wang, Shuang Lin, Xiao-Nian Li, Hucheng Zhu, Zengwei Luo, Yongbo Xue, Zhengxi Hu, Yonghui Zhang

**Affiliations:** 1 Hubei Key Laboratory of Natural Medicinal Chemistry and Resource Evaluation, School of Pharmacy, Tongji Medical College, Huazhong University of Science and Technology, Wuhan 430030, China; yangbeiye123@163.com (B.Y.); weiguang_s@hust.edu.cn (W.S.); jpwang1001@163.com (J.W.); 13207186519@163.com (S.L.); zhuhucheng0@163.com (H.Z.); luozengwei@gmail.com (Z.L.); yongboxue@mail.hust.edu.cn (Y.X.); 2 State Key Laboratory of Phytochemistry and Plant Resources in West China, Kunming Institute of Botany, Chinese Academy of Sciences, Kunming 650204, China; lixiaonian@mail.kib.ac.cn

**Keywords:** marine-derived fungi, *Penicillium* sp. TJ403-1, breviane spiroditerpenoid, IDH1 inhibitory activity, cytotoxicity

## Abstract

Marine-derived fungi are a promising and untapped reservoir for discovering structurally interesting and pharmacologically active natural products. In our efforts to identify novel bioactive compounds from marine-derived fungi, four breviane spiroditerpenoids, including a new compound, brevione O (**1**), and three known compounds breviones I (**2**), J (**3**), and H (**4**), together with a known diketopiperazine alkaloid brevicompanine G (**5**), were isolated and identified from an ethyl acetate extract of the fermented rice substrate of the coral-derived fungus *Penicillium* sp. TJ403-1. The absolute structure of **1** was elucidated by HRESIMS, one- and two-dimensional NMR spectroscopic data, and a comparison of its electronic circular dichroism (ECD) spectrum with the literature. Moreover, we confirmed the absolute configuration of **5** by single-crystal X-ray crystallography. All the isolated compounds were evaluated for isocitrate dehydrogenase 1 (IDH1) inhibitory activity and cytotoxicity, and compound **2** showed significant inhibitory activities against HL-60, A-549, and HEP3B tumor cell lines with IC_50_ values of 4.92 ± 0.65, 8.60 ± 1.36, and 5.50 ± 0.67 µM, respectively.

## 1. Introduction

Since the ocean covers over 70% of the Earth’s surface, marine organisms are regarded as a prolific and under-explored resource of bioactive natural products [1,2]. Over the past few decades, with the discovery of plenty of new chemicals, marine-derived fungi have increasingly attracted the attention of natural product chemists and biologists, largely due to their surprising potentials for drug discovery [3,4,5].

Breviane spiroditerpenoids, which are biosynthesized from geranylgeranyl and pyrone derived from three molecules of acetyl CoA and one methyl from methionine, are an important group of architecturally complex and bioactive meroterpenoids [6]. Since the first breviane spiroditerpenoid, namely brevione A, was found from *Penicillium brevicompactum* Dierckx in 2000 [7], a total of 14 naturally occurring compounds with identical skeletons, showing intriguing allelopathic, anti-HIV, cytotoxic, and A*β* aggregate-induced neurotoxic inhibitory effects have been reported to date [7,8,9,10,11]. Notably, their architecturally complex frameworks with multiple chiral centers and attractive biological profiles made these natural products target molecules for total synthesis [12,13,14].

**Figure 1:**
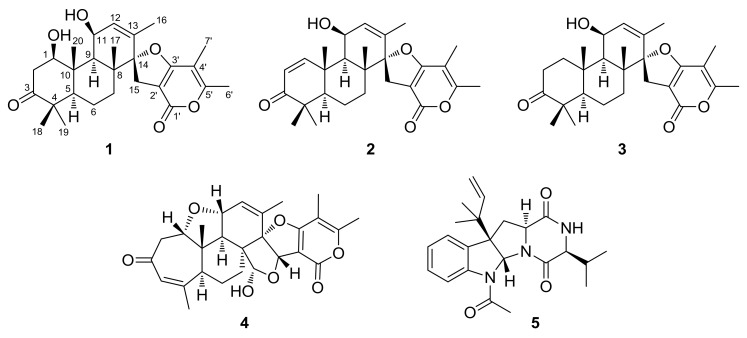
Structures of compounds **1**–**5**.

Previously, we performed a chemical investigation on a mangrove-derived fungus, *Daldinia eschscholzii*, resulting in the isolation and identification of three novel polyketide glycosides, daldinisides A–C, and two new alkaloids, 1-(3-indolyl)-2*R*,3-dihydroxypropan-1-one and 3-ethyl-2,5-pyrazinedipropanoic acid [15]. Interestingly, daldinisides A–C represented a rare class of d-ribose-containing natural products. In our continuous exploration for structurally unique and biologically active natural products from the marine-derived fungi, our attention was focused on the coral-derived fungi, a neglected and insufficiently explored natural resource. A chemical investigation of the fermented rice substrate of the coral-derived fungus *Penicillium* sp. TJ403-1 afforded four breviane spiroditerpenoids, including a new compound, brevione O (**1**), and three known compounds breviones I (**2**), J (**3**), and H (**4**), together with a known diketopiperazine alkaloid brevicompanine G (**5**). Herein, the details of the isolation, structural elucidation, and bioactivity evaluations of these compounds (Figure 1) are reported.

## 2. Results

**Figure 2:**
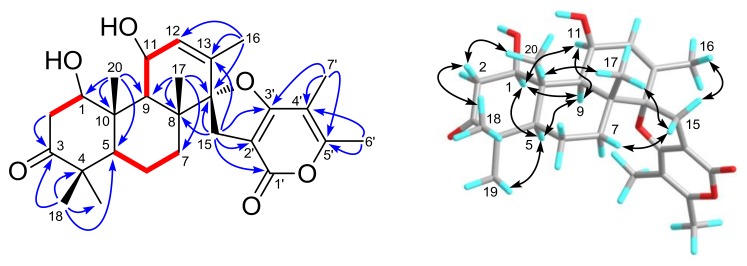
Selected ^1^H–^1^H COSY (red lines), HMBC (blue arrows), and NOESY (black arrows) correlations of compound **1**.

Brevione O (**1**), purified as a white powder, was assigned the molecular formula C_27_H_36_O_6_, based on the HRESIMS *m*/*z* 479.2382 [M + Na]^+^ (calcd. for C_27_H_36_O_6_Na, 479.2410) and ^13^C-NMR analysis (see Supplementary Materials), corresponding to 10 degrees of unsaturation. Its ^1^H-NMR data (Table 1) showed signals of seven methyl groups (*δ*_H_ 1.76 (d, *J* = 1.6 Hz, H_3_-16), 1.21 (s, H_3_-17), 1.09 (s, H_3_-18), 1.03 (s, H_3_-19), 1.48 (s, H_3_-20), 1.92 (s, H_3_-6′), and 2.25 (s, H_3_-7′)), one olefinic proton (*δ*_H_ 5.74 (dd, *J* = 1.6, 4.8 Hz, H-12)), and two oxygenated methine protons (*δ*_H_ 3.95 (dd, *J* = 6.2, 9.2 Hz, H-1) and 4.68 (m, H-11)). Detailed analysis of the ^13^C- and DEPT NMR spectroscopic data (Table 1) of **1** indicated the presence of seven sp^3^ methyls, four sp^3^ methylenes, five methines (including two oxygenated and one olefinic), eleven non-protonated carbons (including one oxygenated, five olefinic, one ester carbonyl, and one ketone). Comparing the HRESIMS and NMR data with previously reported C_27_ meroterpenoids from the *Penicillium* species [7,8,9,10,11], these data were indicative of a breviane spiroditerpenoid, and interpretations of the ^1^H–^1^H COSY and HMBC spectra (Figure 2) of **1** established its planar structure as the C-1 hydroxylated analogue of the known compound brevione J (**3**), a co-isolated known metabolite that was initially characterized from a marine-derived *Penicillium* sp. [10].

In the NOESY experiment (Figure 2), the cross-peaks of H_3_-18/H-2*β* (*δ*_H_ 2.81), H-2*β*/H_3_-20, H_3_-20/H_3_-17, and H_3_-19/H-5, H-5/H-9, H-9/H-11, and correlations of H-1 with H-5, H-9, and H-11 indicated that H_3_-17, H_3_-18, and H_3_-20 were all *β*-oriented, while H-1, H-5, H-9, H-11, and H_3_-19 were all on the opposite side with *α*-orientations. Moreover, the NOE correlations of H_2_-15 with H-7*β* (*δ*_H_ 1.62), H_3_-16, and H_3_-17 indicated that two planes of C- and D-rings were vertically arranged and that C-15 was on the upside of C-ring. Thus, the relative configuration of compound **1** was established.

**Figure 3:**
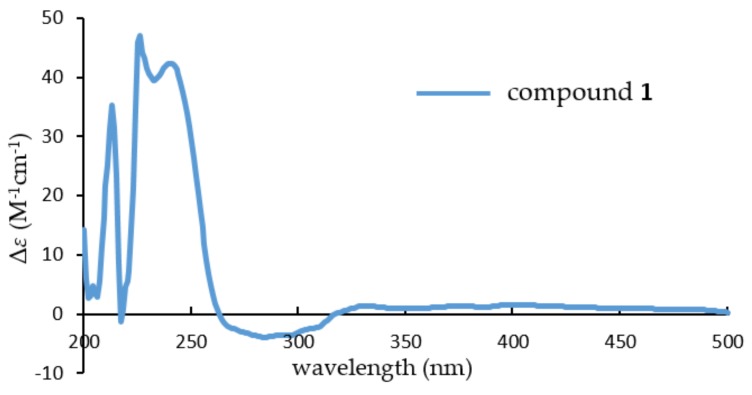
Experimental electronic circular dichroism (ECD) spectrum of compound **1**.

To determine its absolute stereochemistry, the experiment electronic circular dichroism (ECD) spectrum (Figure 3) of **1**, which was measured in MeOH, was compared to that of brevione J (**3**). Their nearly identical ECD spectra indicated that the absolute stereochemistry of **1** should be identified as 1*R*,5*R*,8*R*,9*R*,10*S*,11*S*,14*S*.

**Figure 4:**
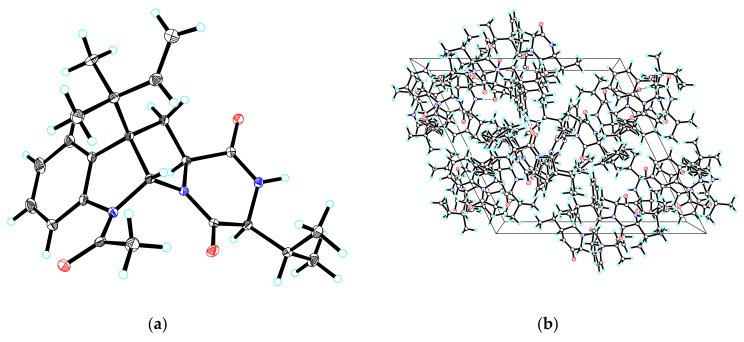
(**a**) ORTEP drawing of compound **5**; (**b**) View of the pack drawing of **5** and hydrogen-bonds are shown as dashed lines.

By comparing their specific rotation and NMR data with the literature, compounds **2**–**5** were identified as breviones I (**2**) [10], J (**3**) [10], H (**4**) [9], and brevicompanine G (**5**) [16], respectively. Remarkably, the absolute configuration of **5** was first confirmed via single-crystal X-ray crystallography with a Flack parameter = −0.03(4) (Figure 4).

Isocitrate dehydrogenase 1 (IDH1) catalyzes the oxidative decarboxylation of isocitrate to α-ketoglutarate (α-KG). Neomorphic mutations in IDH1, mostly occurring at arginine 132, are frequently found in several human cancer types, including glioma, acute myeloid leukemia (AML), and myeloproliferative neoplasm [17]. When our research group screened for the IDH1 inhibitors from our natural product libraries, all the isolates **1**–**5** were evaluated for IDH1 inhibitory activity; unfortunately, none of them was active at a concentration of 20 μM. Additionally, compounds **1**–**5** were investigated for cytotoxic activities against several human tumor cell lines, including HL-60 (acute leukemia), MM231 (breast cancer), A-549 (lung cancer), HEP3B (hepatic cancer), SW-480 (colon cancer), and one normal colonic epithelial cell, NCM460. As shown in Table 2, compound **2**, with no cytotoxic activity against the NCM460 cell, showed significant inhibitory potency against the HL-60, A-549, and HEP3B cell lines, with IC_50_ values of 4.92 ± 0.65, 8.60 ± 1.36, and 5.50 ± 0.67 µM, respectively. However, other compounds showed no obvious cytotoxicity against all these cell lines. Comparison of the IC_50_ values of **2** and **3** implied that the *α*,*β*-unsaturated carbonyl group plays an essential role in the cytotoxic activity. This group might interact with biological molecules by forming covalent bonds with free thiol of cysteine [18]; thus, the potential mechanism deserves further investigation in a following study.

## 3. Materials and Methods

### 3.1. General Experimental Procedures

Optical rotations, ultraviolet (UV), and ECD data were recorded on a PerkinElmer 341 polarimeter (Waltham, MA, USA), a Varian Cary 50 (Santa Clara, CA, USA), and a JASCO-810 CD (Oklahoma City, OK, USA) spectrometer instrument, respectively. FT-IR spectra were measured by using a Bruker Vertex 70 instrument (Billerica, MA, USA). One- and two-dimensional NMR data were collected from a Bruker AM-400 instrument (Bruker, Karlsruhe, Germany). The ^1^H- and ^13^C-NMR chemical shifts of the solvent peaks for methanol-*d*_4_ (*δ*_H_ 3.31 and *δ*_C_ 49.0) were referenced. The positive ion mode on a Thermo Fisher LC-LTQ-Orbitrap XL instrument (Waltham, MA, USA) was used for the measurement of high-resolution electrospray ionization mass spectra (HRESIMS). Semi-preparative HPLC separations were performed on an Agilent 1200 instrument with a reversed-phased C_18_ column (5 μm, 10 × 250 mm), using a UV detector or a Dionex HPLC system (Sunnyvale, CA, USA) which was equipped with an Ultimate 3000 autosampler injector, an Ultimate 3000 pump, and an Ultimate 3000 DAD controlled by Chromeleon software (version 6.80). Column chromatography (CC) was performed using silica gel (200−300 mesh, Qingdao Marine Chemical, Inc., Qingdao, China), Sephadex LH-20 (GE Healthcare Bio-Sciences AB, Uppsala, Sweden), and Lichroprep RP-C_18_ gel (40–63 μm, Merck, Darmstadt, Germany). Thin-layer chromatography was performed with the silica gel 60 F_254_ and RP-C_18_ F_254_ plates (Merck, Darmstadt, Germany), and spots were visualized by spraying heated silica gel plates with 10% H_2_SO_4_ in EtOH.

### 3.2. Fungal Material

Strain TJ403-1 was isolated from a piece of the inner tissues of a fresh soft coral of the genus *Alcyonium* (an unidentified sp.), which was collected from the Sanya Bay, Hainan Island, China. According to its morphology and sequence analysis of the ITS (Internal Transcribed Spacer) region of the rDNA, the strain was identified as *Penicillium* sp. and its sequence data have been submitted to the GenBank with the accession no. MG839539. This strain has been reserved in the culture collection of Tongji Medical College, Huazhong University of Science and Technology (Wuhan, China).

### 3.3. Cultivation, Extraction, and Isolation

The TJ403-1 strain was incubated on potato dextrose agar (PDA) at 28 °C for 6 days in a stationary phase to prepare the seed cultures, which were then cut into small pieces (about 0.4 × 0.4 × 0.4 cm) and inoculated into 350 × 500 mL sterilized Erlenmeyer flasks (each comprised of 200 g rice and 200 mL distilled water). After an incubation at 28 °C for 40 days, 300 mL EtOAc was added to all flasks to stop the growth of cells. Then, the filtrates were collected, and the fermented rice substrate was extracted with EtOAc (8 × 30 L) six times. Lastly, these two parts of the extracts were mixed together. Under reduced pressure, the organic solvent was evaporated to dryness to obtain a dark brown crude extract (700 g).

The EtOAc extract (700 g) was subjected to silica gel CC eluted with a gradient of petroleum ether/EtOAc/MeOH (stepwise 20:1:0 to 1:1:1, *v*/*v*/*v*) to afford eight fractions (Fr.1–Fr.8). Fr.5 was separated through RP-C_18_ CC (MeOH/H_2_O, 20% to 100%, *v*/*v*) to give five subfractions (Fr.5.1–Fr.5.5). Then, Fr.5.3 was purified via silica gel CC, eluted with CH_2_Cl_2_/MeOH (stepwise 50:1 to 30:1, *v*/*v*) to afford two additional subfractions (Fr.5.3.1−5.3.2). Further separations of Fr.5.3.1 through Sephadex LH-20 eluted with a mixture of CH_2_Cl_2_/MeOH (1:1, *v*/*v*) and repeated semi-preparative HPLC (MeOH/H_2_O, 70:30, *v*/*v*; 2 mL/min) yielded compounds **2** (16 mg), **3** (8 mg), and **5** (9 mg). Fr.6 was loaded onto RP-C_18_ CC (MeOH/H_2_O, 20% to 100%, *v*/*v*) to give five subfractions (Fr.6.1–Fr.6.5). By using the silica gel CC (CH_2_Cl_2_/MeOH, 40:1, *v*/*v*) and repeated semi-preparative HPLC (MeOH/H_2_O, 60:40, *v*/*v*; 2 mL/min) methods, Fr.6.3 was finally separated to yield compounds **1** (5 mg) and **4** (60 mg).

*Brevione O* (**1**): white powder; [*α*${]}_{D}^{23}$: +94.1 (*c* 0.14, MeOH); UV (MeOH) *λ*_max_ (log *ε*): 218 (4.33), 253 (3.73), 307 (3.46) nm; CD (MeOH) *λ*_max_ (Δ*ε*): 213 (+35.24), 225 (+45.98), 238 (+41.91), 283 (–3.94); IR (KBr) *ν*_max_: 3423, 2925, 2855, 1703, 1573, 1446, 1388, 1363, 1275, 1116, 1062, 983, 924, 856, 746 cm^−1^; HRESIMS *m*/*z* 479.2382 [M + Na]^+^ (calcd. for C_27_H_36_O_6_Na, 479.2410). For ^1^H- and ^13^C-NMR data, see Table 1.

### 3.4. X-ray Crystallographic Analysis

At room temperature, after attempts with various organic solvents, compound **5** was obtained as colorless crystals from MeOH/H_2_O (15:1, *v*/*v*) by slow evaporation. The intensity data for compound **5** was collected with a Bruker APEX DUO diffractometer, which was outfitted with an APEX II CCD by using graphite-monochromated Cu K*α* radiation (100 K). The Bruker SAINT was used for the cell refinement and data reduction of compound **5**, whose structure was then solved and refined through direct means by using SHELXS-97 program (Göttingen, Germany) [19]. The crystallographic data for compound **5** have been deposited in the Cambridge Crystallographic Data Center (CCDC 1827348 for **5**). Copies of the data can be obtained free of charge from the CCDC, 12 Union Road, Cambridge CB 1EZ, UK (fax: Int. +44(0) (1223) 336 033); e-mail: deposit@ccdc.cam.ac.uk).

Crystallographic data for brevicompanine G (**5**): C_23_H_29_N_3_O_3_, *M* = 395.49, *a* = 18.2192(5) Å, *b* = 13.1497(3) Å, *c* = 19.6255(5) Å, *α* = 90°, *β* = 116.2910(10)°, *γ* = 90°, *V* = 4215.44(19) Å^3^, *T* = 100(2) K, space group *P*21, *Z* = 8, *μ*(CuK*α*) = 0.668 mm^−1^, 38,513 reflections measured, 14,878 independent reflections (*R_int_* = 0.0360). The final *R*_1_ values were 0.0364 (*I* > 2*σ*(*I*)). The final *wR*(*F*^2^) values were 0.0949 (*I* > 2*σ*(*I*)). The final *R*_1_ values were 0.0366 (all data). The final *wR*(*F*^2^) values were 0.0952 (all data). The goodness of fit on *F*^2^ was 1.045. Flack parameter = −0.03(4).

### 3.5. In Vitro IDH1(R132H) Inhibition Assay

The activity and inhibition of IDH1 (R132H) was determined by measuring the initial linear consumption of NADPH of the reaction. The enzyme activity assay was carried out in a 96-well microplate, using the purified IDH1 (R132H) protein in buffer solution containing 50 mM HEPES pH = 7.5, 4 mM MgCl_2_, 100 mM NaCl, and 0.1 mg/mL bovine serum albumin. For inhibition assay, triplicate samples of compounds (10 µL) were incubated with the protein (2 µg/mL, 20 µL) and 60 µL buffer for 5 min. The reaction was initiated by adding 2 mM α-KG, 100 μM NADPH (10 µL) into the 96-well microplate. The consumption of NADPH was measured by monitoring the optical absorbance of each well every 30 s at 340 nm, which was the maximum absorption wavelength of NADPH, using a Biotek Synergy HT microplate reader.

### 3.6. Cytotoxicity Assay

Five human tumor cell lines (HL-60, MM231, A-549, HEP3B, and SW480), together with one non-cancerous cell line, the human normal colonic epithelial cell NCM460, were used in the cytotoxic activity assay. All cells were cultured in DMEM or RPMI-1640 medium (HyClone, Logan, UT, USA), supplemented with 10% fetal bovine serum (HyClone) at 37 °C in a humidified atmosphere with 5% CO_2_. The cell survival assay was performed using the MTT method. Briefly, 100 μL suspended cells at an initial density of 1 × 10^5^ cells/mL were seeded into each well of the 96-well culture plates and allowed to adhere for 12 h before addition of the test compounds. Each tumor cell line was exposed for 48 h to the test compounds at concentrations ranging from 0.0625 to 40 μM, with DDP (*cis*-platin, Sigma, St. Louis, MO, USA) and paclitaxel as positive controls. After incubation, culture supernatants were removed and exchanged with medium containing 0.5 mg/mL MTT. Then, after 4 h incubation in darkness at 37 °C, the medium was removed, and cells were added with 100 μL dimethyl sulfoxide. The absorbance at 570 nm was measured and data are expressed as averages of three replicates. The value of inhibition was calculated by using the following formula: % inhibition = (1 − OD_treated_/OD_control_) × 100. The IC_50_ values were calculated by using a standard dose-response curve fitting with Prism (version 5.0, GraphPad Software, La Jolla, CA, USA).

## 4. Conclusions

To sum up, four breviane spiroditerpenoids (**1**–**4**), including a new compound, brevione O (**1**), and three known compounds breviones I (**2**), J (**3**), and H (**4**), together with a known diketopiperazine alkaloid (**5**), were isolated and identified from an ethyl acetate extract of the fermented rice substrate of the coral-derived fungus *Penicillium* sp. TJ403-1. The absolute structure of **1** was elucidated on the basis of HRESIMS, one- and two-dimensional NMR spectroscopic data, and a comparison of its ECD spectrum with data gathered from the literature. Remarkably, despite the fact that the structure of brevicompanine G (**5**) was once established via a combination of NMR spectroscopic data and biosynthetic logic-based consideration, our current work is the first to confirm the absolute configuration of **5** by single-crystal X-ray crystallography, which will be of great significance for the structure elucidation of complex diketopiperazine alkaloids. Remarkably, compound **2** showed significant inhibitory activities against HL-60, A-549, and HEP3B tumor cell lines, with IC_50_ values of 4.92 ± 0.65, 8.60 ± 1.36, and 5.50 ± 0.67 µM, respectively.

## Figures and Tables

**Table 1:** ^1^H- and ^13^C-NMR data for brevione O (**1**) in methanol-*d*_4_ (*δ* in ppm, *J* in Hz).

No.	1	
	*δ*_H_ *^a^*^,*b*^	*δ*_C_ *^c^*
1	3.95 dd (6.2, 9.2)	78.4 CH
2	2.64 dd (6.2, 14.6); 2.81 dd (9.2, 14.6)	45.0 CH_2_
3	-	216.2 C
4	-	48.5 C
5	1.20 m	53.8 CH
6	1.66 m; 1.91 m	20.1 CH_2_
7	1.39 m; 1.62 m	32.5 CH_2_
8	-	41.7 C
9	1.78 d (5.7)	52.6 CH
10	-	44.8 C
11	4.68 m	67.6 CH
12	5.74 dd (1.6, 4.8)	131.1 CH
13	-	134.0 C
14	-	101.1 C
15	3.05 s	30.1 CH_2_
16	1.76 d (1.6)	19.0 CH_3_
17	1.21 s	19.1 CH_3_
18	1.09 s	21.7 CH_3_
19	1.03 s	26.1 CH_3_
20	1.48 s	14.4 CH_3_
1′	-	173.5 C
2′	-	100.6 C
3′	-	164.2 C
4′	-	104.8 C
5′	-	162.2 C
6′	1.92 s	9.5 CH_3_
7′	2.25 s	17.2 CH_3_

*^a^* Recorded at 400 MHz; *^b^* “m” means overlapped or multiplet with other signals; *^c^* Recorded at 100 MHz.

**Table 2:** Cytotoxic activities of compounds **1**–**5** against human tumor cell lines.

Compound	IC_50_ (µM)					
	HL-60	MM231	A-549	HEP3B	SW480	NCM460
**1**	>40	>40	>40	>40	>40	>40
**2**	4.92 ± 0.65	>40	8.60 ± 1.36	5.50 ± 0.67	21.17 ± 2.72	>40
**3**	25.63 ± 3.84	>40	>40	>40	>40	>40
**4**	>40	>40	>40	>40	>40	>40
**5**	21.77 ± 3.48	>40	18.41 ± 2.15	>40	>40	>40
*Cis*-platin ^1^	1.63 ± 0.11	3.84 ± 0.25	2.79 ± 0.36	2.96 ± 0.22	1.42 ± 0.11	0.85 ± 0.07
Paclitaxel ^1^	<0.008	<0.008	<0.008	<0.008	<0.008	<0.008

^1^ *Cis*-platin and paclitaxel were used as positive controls.

## Supplementary Materials

The following are available online at http://www.mdpi.com/1660-3397/16/4/110/s1, 1D (^1^H- and ^13^C-) and 2D (HMBC, HSQC, COSY, NOESY) NMR, HRESIMS, IR, and UV spectra of **1**.

## References

[1] Sueyoshi K., Yamano A., Ozaki K., Sumimoto S., Iwasaki A., Suenaga K., Teruya T. (2017). Three new malyngamides from the marine cyanobacterium *Moorea producens*. Mar. Drugs.

[2] Imhoff J.F. (2016). Natural products from marine fungi—Still an underrepresented resource. Mar. Drugs.

[3] Mayer A.M.S., Glaser K.B., Cuevas C., Jacobs R.S., Kem W., Little R.D., McIntosh J.M., Newman D.J., Potts B.C., Shuster D.E. (2010). The odyssey of marine pharmaceuticals: A current pipeline perspective. Trends Pharmacol. Sci..

[4] Newman D.J., Cragg G.M. (2017). Current Status of marine-derived compounds as warheads in anti-tumor drug candidates. Mar. Drugs.

[5] Cherigo L., Lopez D., Martinez-Luis S. (2015). Marine natural products as breast cancer resistance protein inhibitors. Mar. Drugs.

[6] Geris R., Simpson T.J. (2009). Meroterpenoids produced by fungi. Nat. Prod. Rep..

[7] Macías F.A., Varela R.M., Simonet A.M., Cutler H.G., Cutler S.J., Ross S.A., Dunbar D.C., Dugan F.M., Hill R.A. (2000). (+)-Brevione A. The first member of a novel family of bioactive spiroditerpenoids isolated from *Penicillium brevicompactum* Dierckx. Tetrahedron Lett..

[8] Macías F.A., Varela R.M., Simonet A.M., Cutler H.G., Cutler S.J., Dugan F.M., Hill R.A. (2000). Novel bioactive breviane spiroditerpenoids from *Penicillium brevicompactum* Dierckx. J. Org. Chem..

[9] Li Y., Ye D., Chen X., Lu X., Shao Z., Zhang H., Che Y. (2009). Breviane spiroditerpenoids from an extreme-tolerant *Penicillium* sp. isolated from a deep sea sediment sample. J. Nat. Prod..

[10] Li Y., Ye D., Shao Z., Cui C., Che Y. (2012). A sterol and spiroditerpenoids from a *Penicillium* sp. isolated from a deep sea sediment sample. Mar. Drugs.

[11] Kwon J., Seo Y.H., Lee J.E., Seo E.K., Li S., Guo Y., Hong S.B., Park S.Y., Lee D. (2015). Spiroindole alkaloids and spiroditerpenoids from *Aspergillus duricaulis* and their potential neuroprotective effects. J. Nat. Prod..

[12] Yokoe H., Mitsuhashi C., Matsuoka Y., Yoshimura T., Yoshida M., Shishido K. (2011). Enantiocontrolled total syntheses of breviones A, B, and C. J. Am. Chem. Soc..

[13] Macías F.A., Carrera C., Chinchilla N., Fronczek F.R., Galindo J.C.G. (2010). Synthesis of the western half of breviones C, D, F and G. Tetrahedron.

[14] Takikawa H., Hirooka M., Sasaki M. (2003). The first synthesis of (±)-brevione B, an allelopathic agent isolated from *Penicillium* sp.. Tetrahedron Lett..

[15] Hu Z.X., Xue Y.B., Bi X.B., Zhang J.W., Luo Z.W., Li X.N., Yao G.M., Wang J.P., Zhang Y.H. (2014). Five new secondary metabolites produced by a marine-associated fungus, *Daldinia eschscholzii*. Mar. Drugs.

[16] Du L., Yang X., Zhu T., Wang F., Xiao X., Park H., Gu Q. (2009). Diketopiperazine alkaloids from a deep ocean sediment derived fungus *Penicillium* sp.. Chem. Pharm. Bull..

[17] He Y., Zheng M., Li Q., Hu Z., Zhu H., Liu J., Wang J., Xue Y., Li H., Zhang Y. (2017). Asperspiropene A, a novel fungal metabolite as an inhibitor of cancer-associated mutant isocitrate dehydrogenase 1. Org. Chem. Front..

[18] Sun X., Wang W., Chen J., Cai X., Yang J., Yang Y., Yan H., Cheng X., Ye J., Lu W. (2017). The natural diterpenoid isoforretin A inhibits thioredoxin-1 and triggers potent ROS-mediated antitumor effects. Cancer Res..

[19] Hu Z.X., Liu M., Wang W.G., Li X.N., Hu K., Li X.R., Du X., Zhang Y.H., Puno P.T., Sun H.D. (2018). 7α,20-Epoxy-*ent*-kaurane diterpenoids from the aerial parts of *Isodon pharicus*. J. Nat. Prod..

