# Supplementary material for: A New Breviane Spiroditerpenoid from the Marine-Derived Fungus Penicillium sp. TJ403-1
Source: Mar Drugs. 2018 Mar 29;16(4):110. doi: 10.3390/md16040110 (PMC5923397; doi:10.3390/md16040110)
Supplement: Supplementary file 1 [file marinedrugs-16-00110-s001.pdf]

A New Breviane Spiroditerpenoid from the Marine-Derived Fungus *Penicillium* sp. TJ403-1

Beiye Yang [1,†], Weiguang Sun [1,†], Jianping Wang [1,†], Shuang Lin [1], Xiao-Nian Li [2], Hucheng Zhu [1], Zengwei Luo [1], Yongbo Xue [1], Zhengxi Hu [1,*] and Yonghui Zhang [1,*]

[1]*Hubei Key Laboratory of Natural Medicinal Chemistry and Resource Evaluation, School of Pharmacy, Tongji Medical College, Huazhong University of Science and Technology, Wuhan 430030, People's Republic of China*

[2]*State Key Laboratory of Phytochemistry and Plant Resources in West China, Kunming Institute of Botany, Chinese Academy of Sciences, Kunming 650204, People's Republic of China*

---

* Corresponding author Tel.: +86 027 83692892 (Y.Z.).
E-mail addresses: zhangyh@mails.tjmu.edu.cn (Y.Z.); huzhengxi@hust.edu.cn (Z.H.).

CONTENTS

Figure S1. ^{1}H NMR spectrum of compound **1** (Recorded in methanol-*d*$_4$) 1

Figure S2. ^{13}C NMR spectrum of compound **1** (Recorded in methanol-*d*$_4$) 2

Figure S3. DEPT spectrum of compound **1** (Recorded in methanol-*d*$_4$) 3

Figure S4. HSQC spectrum of compound **1** (Recorded in methanol-*d*$_4$) 4

Figure S5. HMBC spectrum of compound **1** (Recorded in methanol-*d*$_4$) 5

Figure S6. ^{1}H–^{1}H COSY spectrum of compound **1** (Recorded in methanol-*d*$_4$) 6

Figure S7. NOESY spectrum of compound **1** (Recorded in methanol-*d*$_4$) 7

Figure S8. HRESIMS spectrum of compound **1** 8

Figure S9. UV spectrum of compound **1** 8

Figure S10. IR spectrum of compound **1** 9

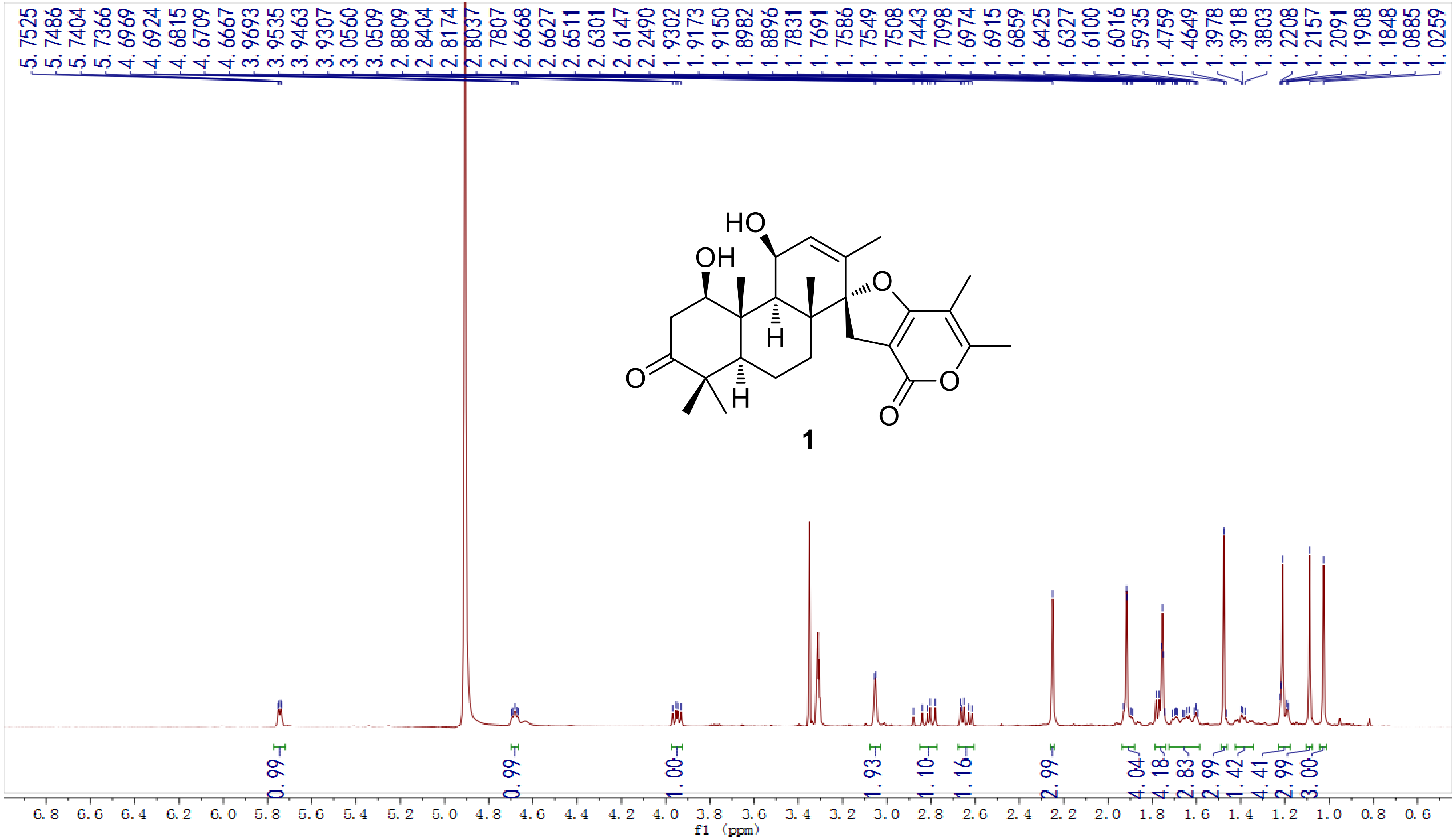

Figure S1. ^{1}H NMR spectrum of compound **1** (Recorded in methanol-d_4)

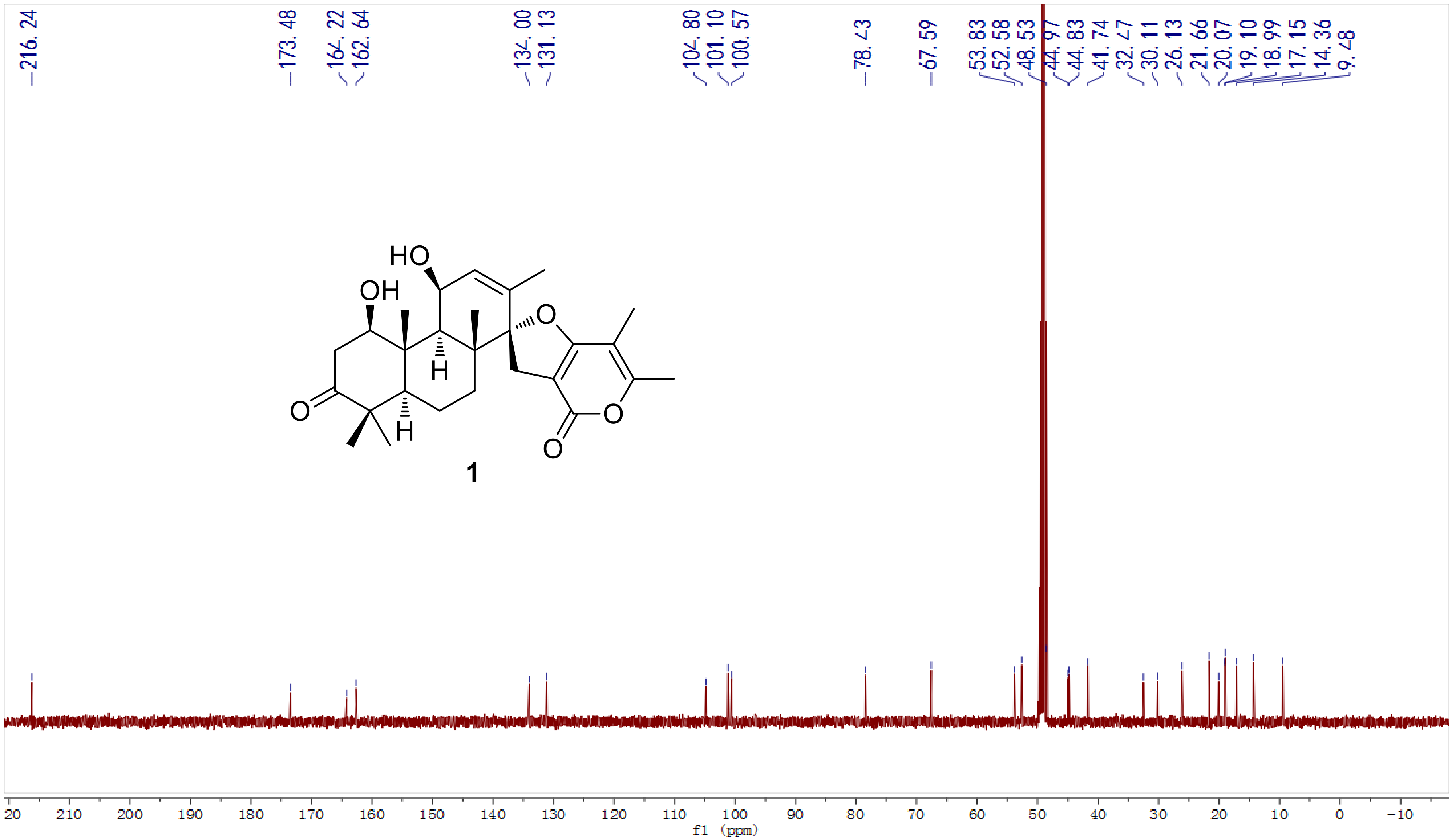

Figure S2. ^{13}C NMR spectrum of compound **1** (Recorded in methanol-d_4)

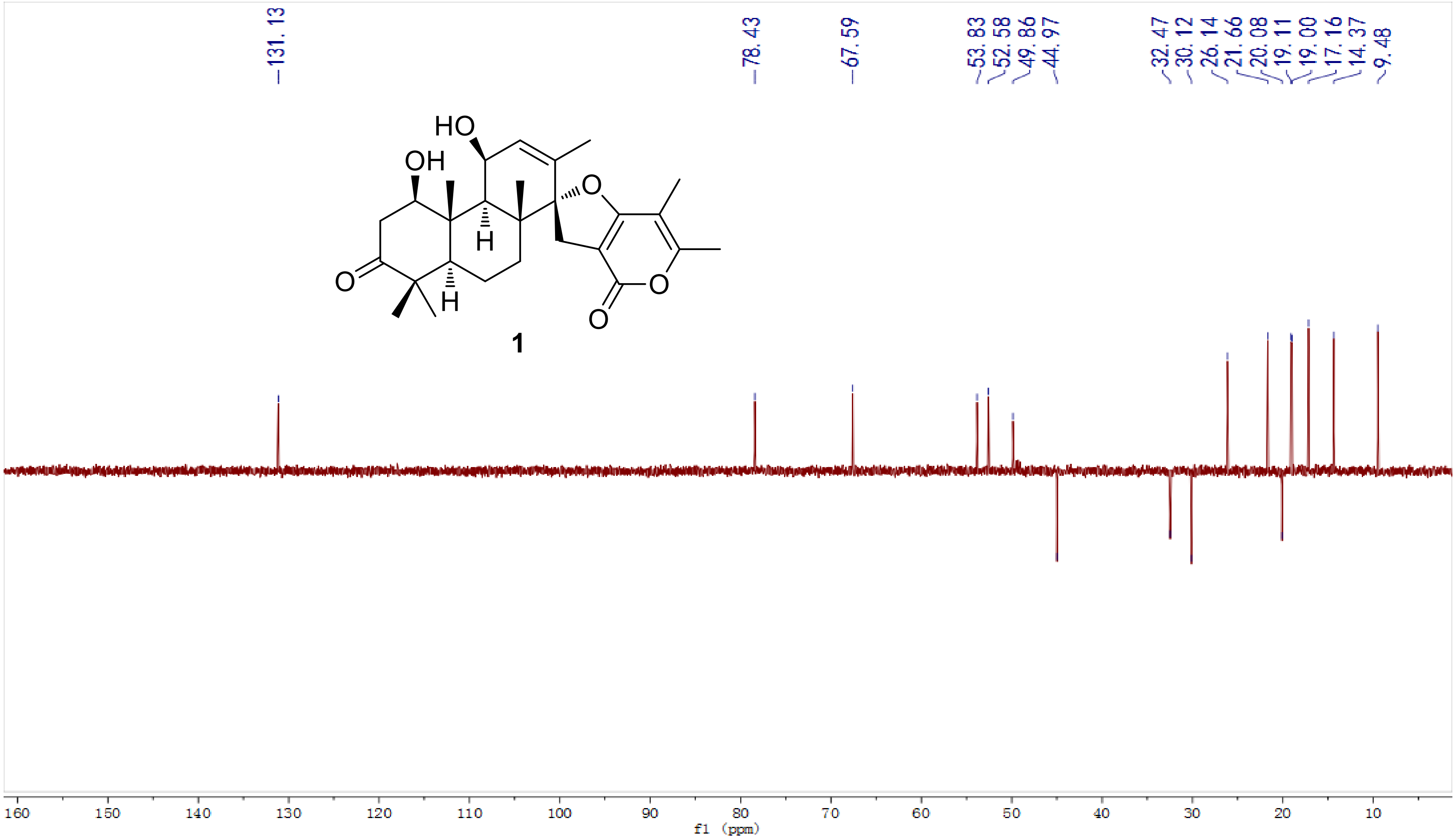

Figure S3. DEPT spectrum of compound **1** (Recorded in methanol-d_4)

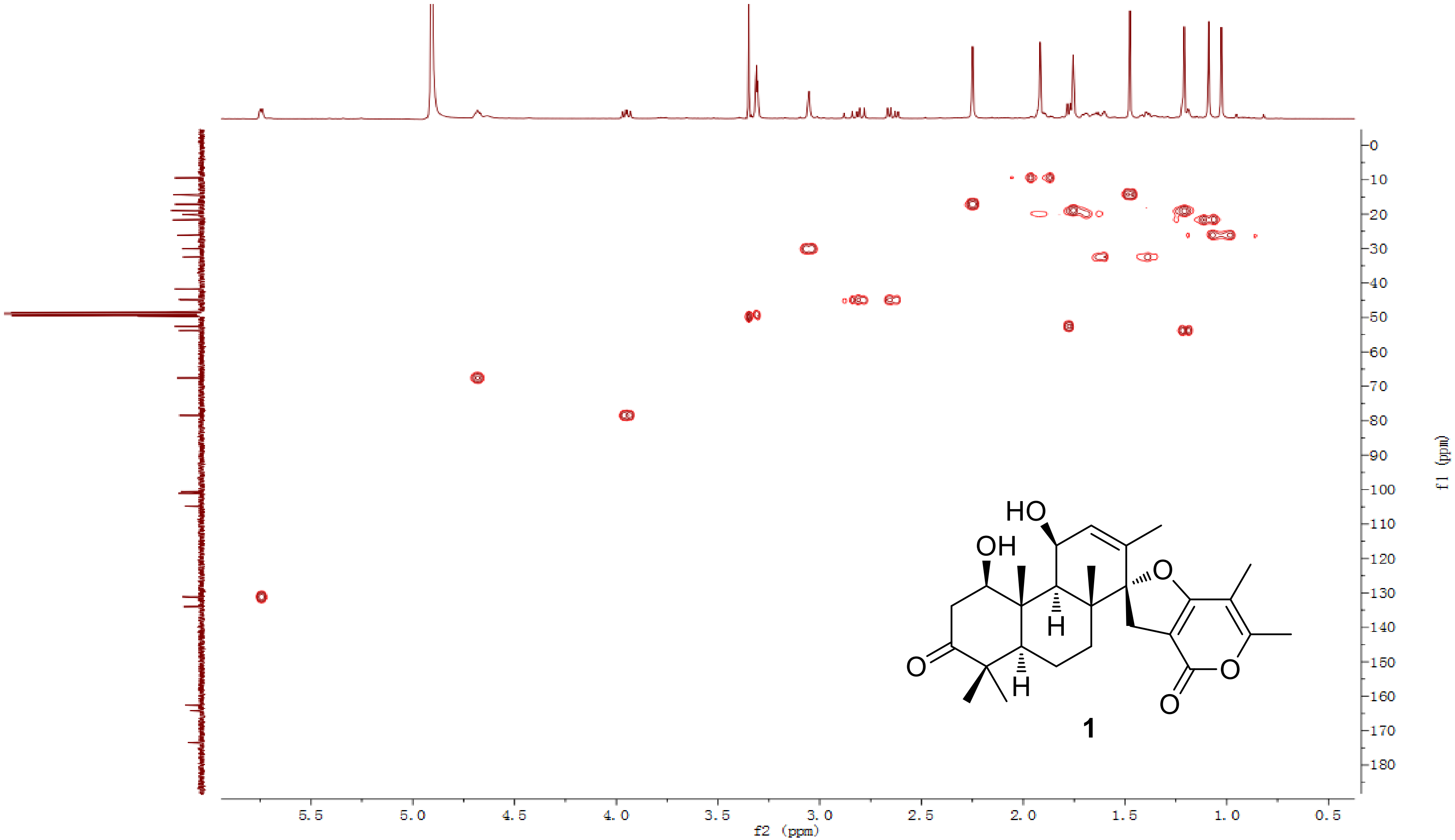

Figure S4. HSQC spectrum of compound **1** (Recorded in methanol-d_4)

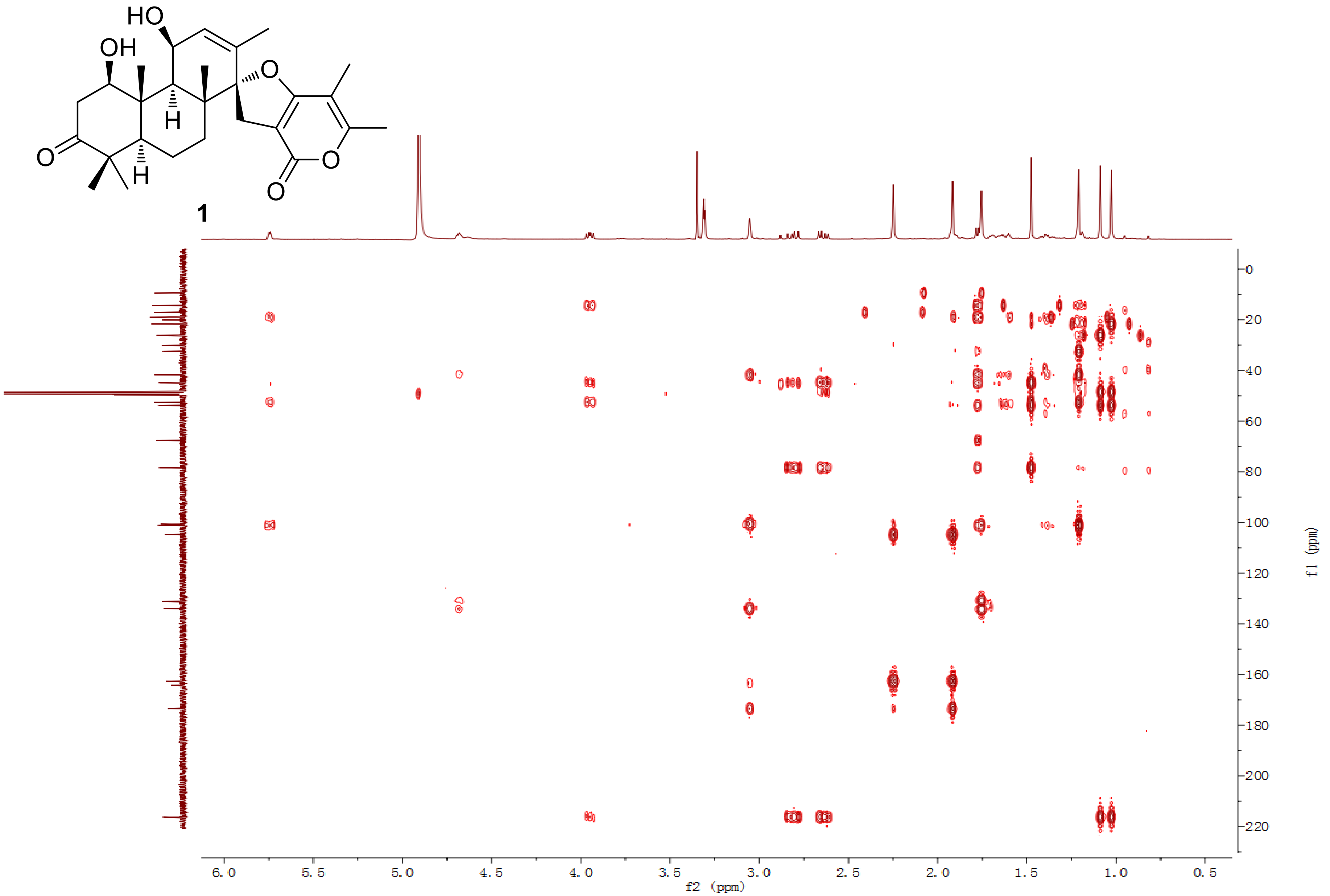

Figure S5. HMBC spectrum of compound **1** (Recorded in methanol-d_4)

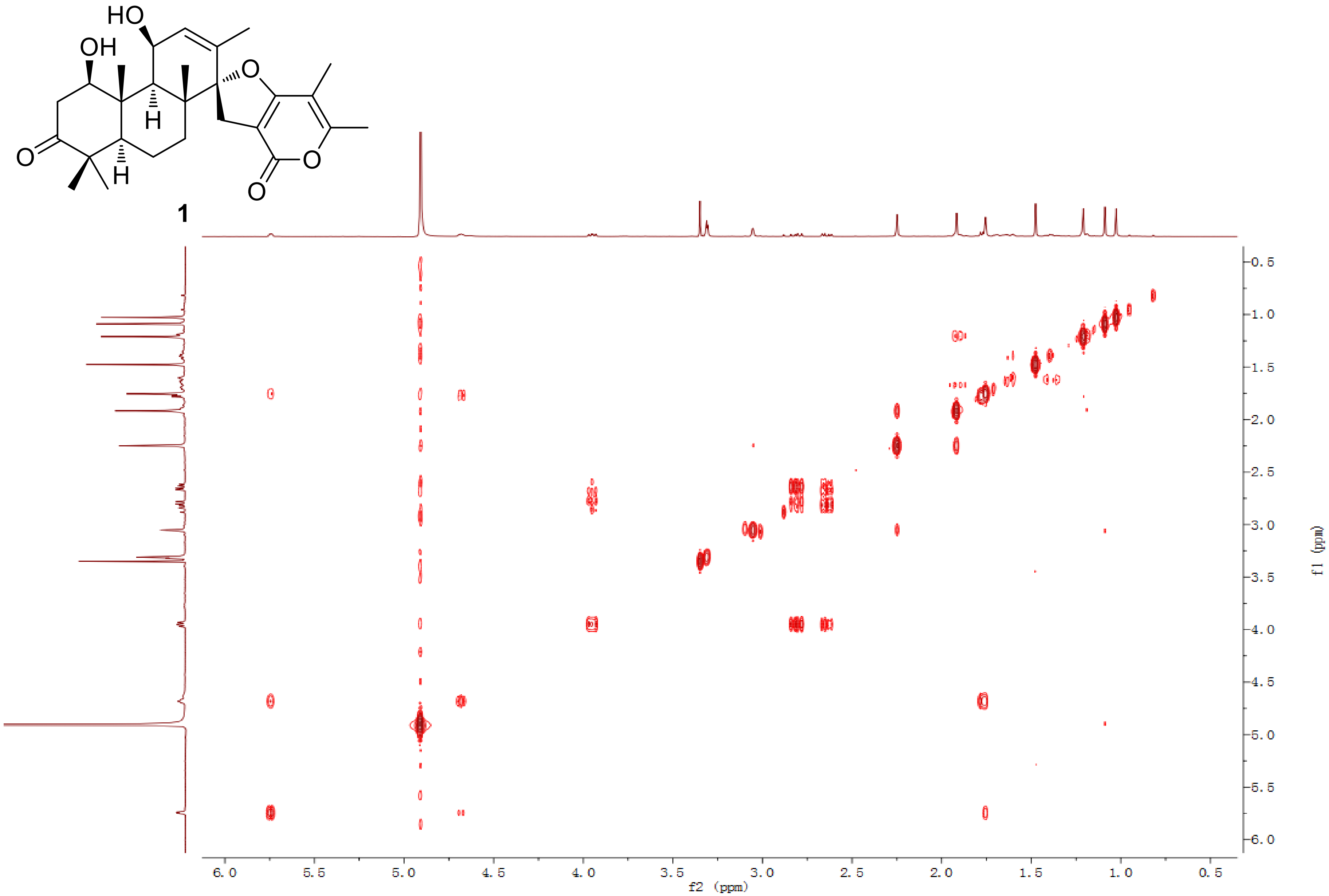

Figure S6. ^{1}H–^{1}H COSY spectrum of compound **1** (Recorded in methanol-d_4)

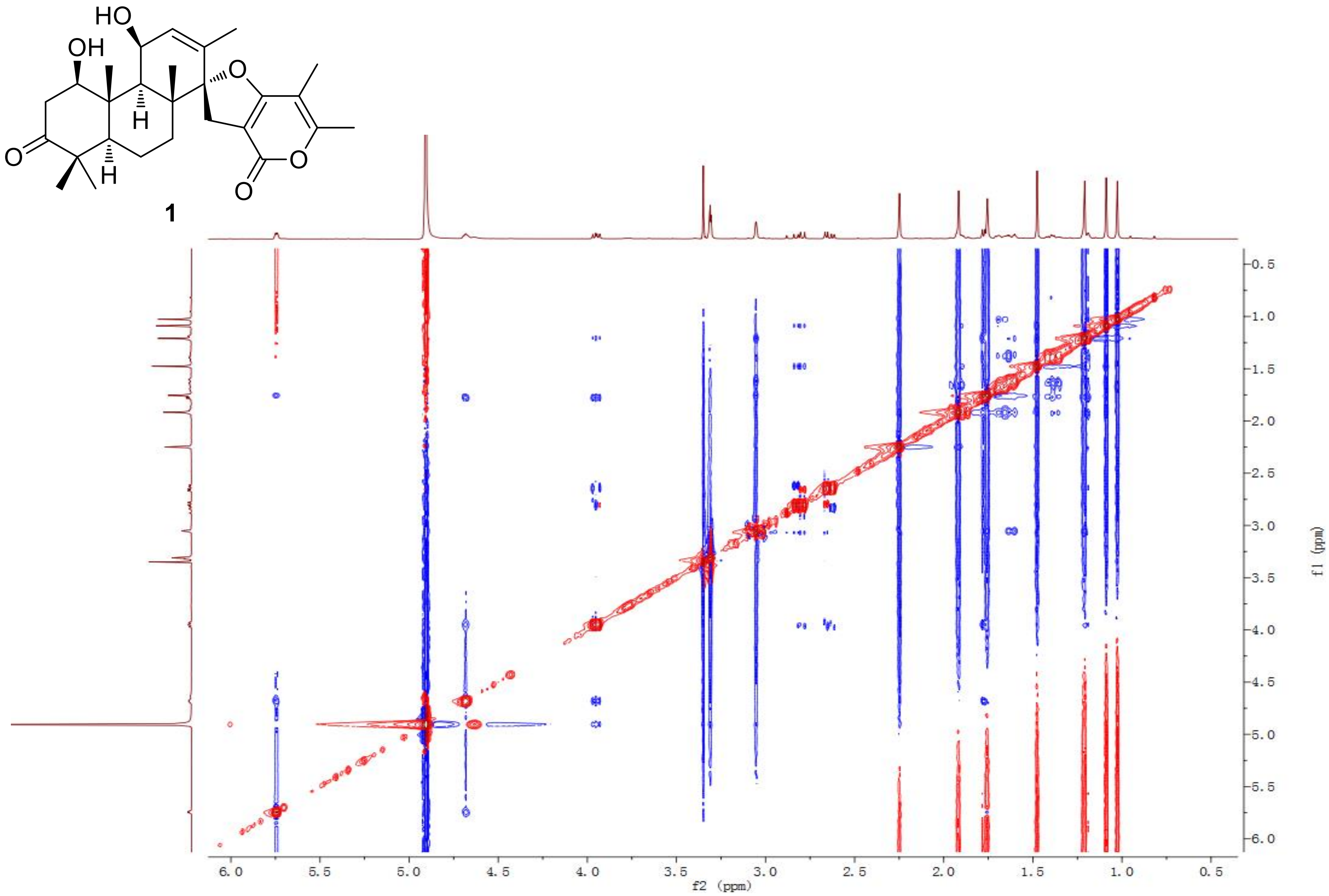

Figure S7. NOESY spectrum of compound **1** (Recorded in methanol-d_4)

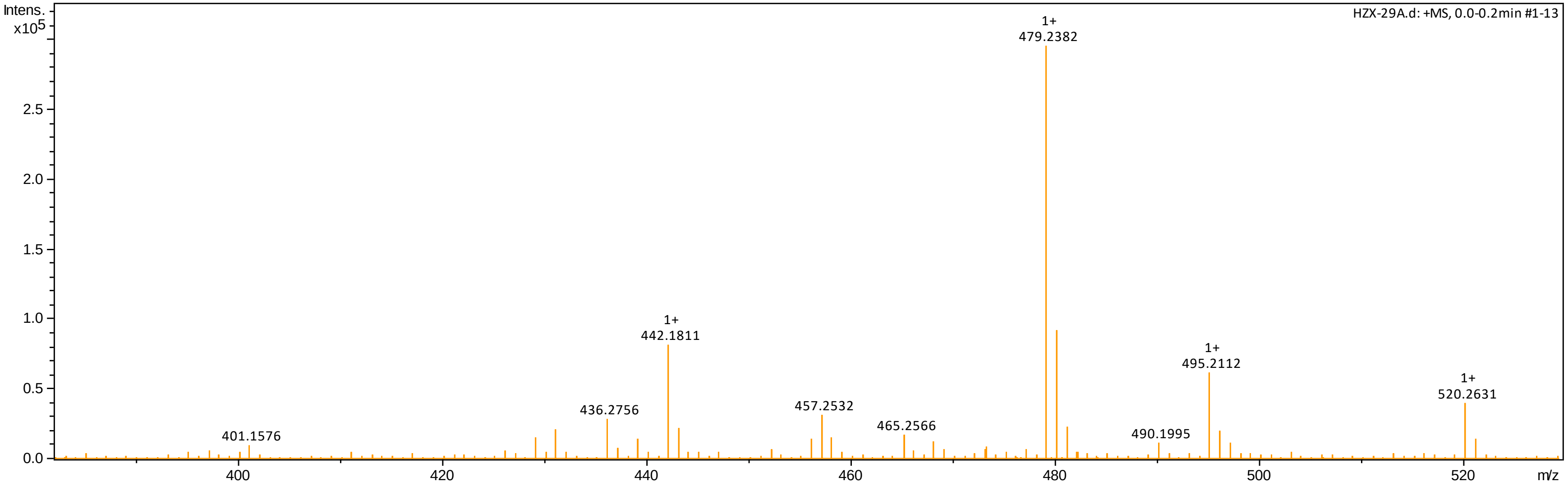

Figure S8. HRESIMS spectrum of compound **1**

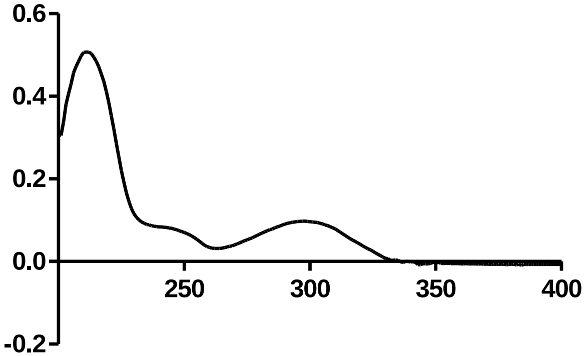

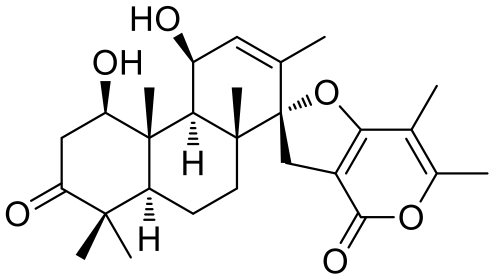

1

Figure S9. UV spectrum of compound **1**

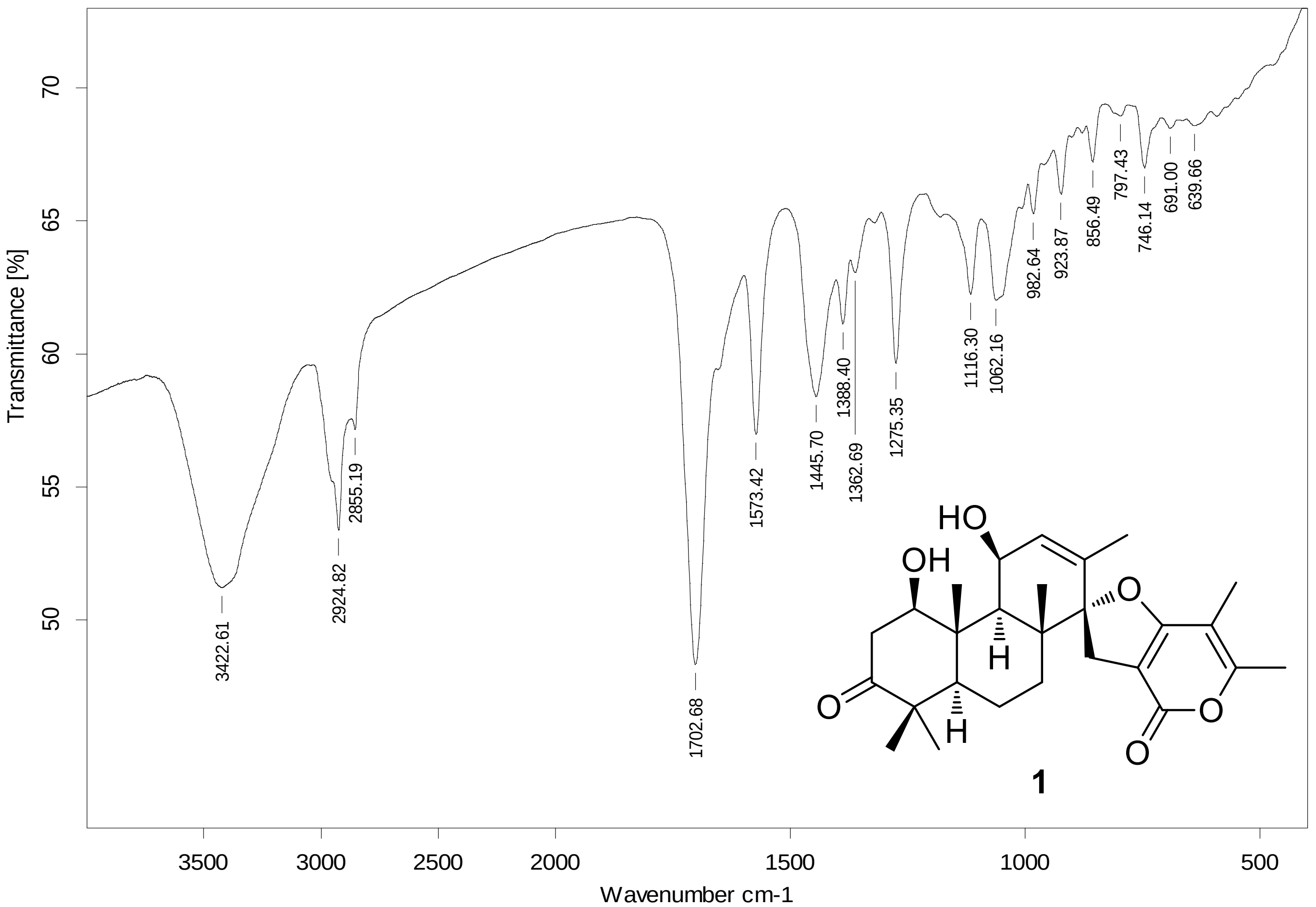

Figure S10. IR spectrum of compound **1**
